# 局限期小细胞肺癌加速超分割放疗同步EP方案化疗的剂量递增Ⅰ期研究

**DOI:** 10.3779/j.issn.1009-3419.2017.01.08

**Published:** 2017-01-20

**Authors:** 静 尤, 会明 于, 马小薇 宋, 晨 石, 晓航 王, 晔 郑, 荣 余, 安辉 石, 广迎 朱

**Affiliations:** 100142 北京，北京大学肿瘤医院暨北京市肿瘤防治研究所，恶性肿瘤发病机制及转化研究教育部重点实验室，放疗科 Key Laboratory of Carcinogenesis and Translational Research (Ministry of Education), Department of Radiation Oncology, Peking University Cancer Hospital & Institute, Beijing 100142, China

**Keywords:** 局限期小细胞肺癌, 同步放化疗, 加速超分割, 化疗剂量递增, Limited stage small cell lung cancer, Concurrent chemo-radiotherapy, Accelerated hyperfractionation, Chemotherapy dose escalation

## Abstract

**背景与目的:**

加速超分割放疗（每日两次方案）联合EP方案同步化疗是美国国立综合癌症网络（National Comprehensive Cancer Network, NCCN）指南推荐的局限期小细胞肺癌的标准治疗方式，但国人对EP方案标准化疗剂量耐受性尚不明确。本研究旨在探讨局限期小细胞肺癌同步放化疗EP方案的最大耐受剂量。

**方法:**

研究纳入病理证实的局限期小细胞肺癌患者，进行加速超分割放疗同步EP方案（依托泊苷+顺铂）化疗，放疗处方剂量为45 Gy/30 f，1.5 Gy/f，每日两次，同一日两次放疗间隔时间≥6 h，5天/周，共3周完成。化疗方案采用依托泊苷联合顺铂，每21天为1周期，具体依托泊苷100 mg/m^2^，d1-d3，顺铂采用剂量递增的方式（第1组为70 mg/m^2^ d1，第2组为75 mg/m^2^ d1）。主要观察指标为治疗期间的血液学毒性。次要观察指标为非血液学毒性和1年总生存期（overall survival, OS）、无进展生存期（progression free survival, PFS）。根据不良事件常用术语评定标准（Common Terminology Criteria for Adverse Events, NCI-CTCAE）4.0，最大耐受剂量设定为6例患者中不超过1例患者出现剂量限制毒性（4级血液学毒性）的剂量，同时下一剂量组6例患者至少2例出现剂量限制性毒性。

**结果:**

研究共纳入20例局限期小细胞肺癌患者，平均年龄49.50（30-68）岁。第1组入组6例患者，1例患者出现4度中性粒细胞减少；后第2组入组14例患者，1例患者出现4度中性粒细胞减少。其中，第1组有4例患者出现≥3度血液学毒性，1例患者出现3度以上放射性食管炎；第2组有10例患者出现≥3度血液学毒性，无患者出现3度以上放射性食管炎。中位随访9.0个月（3.2个月-36.2个月），1年OS、PFS分别为91%、62%。

**结论:**

局限期小细胞肺癌患者采用加速超分割放疗联合EP方案化疗将顺铂剂量递增至75 mg/m^2^是安全的，其有效性还需要进一步扩大样本量和随访更长的时间来证实。

## Abstract (en)

**Background and objective:**

Concurrent twice-daily radiotherapy with chemotherapy of EP regimen is one of the current standard treatments for limited-stage small cell lung cancer. However, the safely tolerated dose of standard chemotherapy for Chinese patients is not decided. This study was to evaluate the toxicity and the maximum tolerated dose (MTD) of etoposide and cisplatin concurrent with thoracic radiation therapy for patients with limited-stage small cell lung cancer.

**Methods:**

Patients with histologically proven limited-stage small cell lung cancer (LS-SCLC) were eligible. The patients underwent thoracic radiotherapy (45 Gy, 1.5 Gy *bid*, 30 fractions for 3 weeks) delivered concurrently with etoposide (100 mg/m^2^ iv, days 1-3) and cisplatin dose escalating from the two levels (70 mg/m^2^ and 75 mg/m^2^ on d1). The primary endpoints were hematologic toxicities during treatment. The secondary endpoints were non-hematologic toxicities, overall survival (OS) and progression-free survival (PFS). According to Common Terminology Criteria for Adverse Events 4.0 (CTC-AE 4.0), maximum tolerant dosage (MTD) was defined as the highest safely tolerated dose at which no more than one patient out of six experiences dose-limiting toxicity (Grades 4 hematologic), with the next higher dose having at least two out of six patients experience dose-limiting toxicity.

**Results:**

From January 2013 to August 2016, 20 patients were enrolled in this study. The median age was 49.5 (30-68). After the first 6 patients were enrolled in Arm 1 (70 mg/m^2^ on d1), one patient had Grade 4 neutropenia. Another 14 patients were enrolled in Arm 2 (75 mg/m^2^ on d1), one patient had Grade 4 neutropenia. The MTD was determined to be etoposide (100 mg/m^2^ iv, d1-d3) and cisplatin dose (75 mg/m^2^ on d1). 4 patients had ≥Grade 3 neutropenia and 1 patients had ≥Grade 3 acute esophagitis in Arm 1. 10 patients had ≥Grade 3 neutropenia and no patient had ≥Grade 3 acute esophagitis in Arm 2. All patients with a median follow-up time was 9.0 months, median OS and PFS were not achieved, 1-year OS and PFS were 91% and 61%, respectively.

**Conclusion:**

The MTD of RT with concurrent chemotherapy of EP regimen for patients with LS-SCLC was etoposide (100 mg/m^2^ iv, d1-d3) and cisplatin dose (75 mg/m^2^ on d1).

同步放化疗联合EP方案化疗是局限期小细胞肺癌（limited-stage small cell lung cancer, LS-SCLC）的标准治疗^[1, 2]^。由于小细胞肺癌倍增时间短，对放化疗敏感，其对放化疗达到较好疗效的比例很高，但小细胞肺癌因其较高的复发率和远处转移率，预后仍较差。

对于LS-SCLC，目前美国国立综合癌症网络（National Comprehensive Cancer Network, NCCN）指南中加速超分割放疗（每日两次方案）联合化疗为Ⅰ类推荐。其中，EP方案推荐剂量为依托泊苷100 mg/m^2^ d1-d3，顺铂80 mg/m^2^ d1，21 d为1个周期。对于广泛期小细胞肺癌化疗的推荐剂量为依托泊苷100 mg/m^2^ d1-d3，顺铂75 mg/m^2^ d1，21 d为1个周期^[3]^，目前国内小细胞肺癌患者内科化疗多采用此剂量，对于LS-SCLC行每日两次放疗联合EP方案化疗，国内临床实践中常用的同步EP方案剂量为依托泊苷100 mg/m^2^ d1-d3，顺铂70 mg/m^2^ d1，21 d为1个周期，但对其耐受性尚不明确。本研究旨在探讨LS-SCLC同步放化疗EP方案的最大耐受剂量（maximum tolerant dosage, MTD）。

## 1 资料与方法

### 1.1 病例选择

入组标准：经组织病理学或细胞学证实的小细胞肺癌患者；根据美国癌症联合会（American Joint Committee on Cancer, AJCC）第7版分期^[4]^为局限期；年龄18岁-70岁；美国东部肿瘤协作组（Eastern Cooperative Oncology Group, ECOG）评分为0分-1分；肝肾功能正常；骨髓功能正常（中性粒细胞≥2.0×10^9^/L，血红蛋白≥100 g/L，血小板≥100×10^9^/L）；肺功能一秒用力呼气容积（forced expiratory volume in one second, FEV_1_）≥1或≥40%预测值；6个月内未出现心肌梗死病史；无心力衰竭或有意义的心律不齐；除皮肤肿瘤外无第二原发肿瘤；既往未行过放化疗。排除标准：病理类型为非小细胞肺癌或混合型；临床分期为广泛期；心肺功能较差，无法耐受同步放化疗；近6个月内出现过心肌梗死病史；因小细胞肺癌既往接受过放化疗或手术治疗。

### 1.2 治疗

治疗前所有患者均签署知情同意书。患者采用同步放化疗方案。化疗方案为EP方案（依托泊苷+顺铂），共4个周期，每21天为1个周期。放疗期间进行2周期EP方案同步化疗。对于病灶较大的患者（肿瘤最大径≥8 cm）先行2个周期诱导化疗。非放疗期间化疗方案为：依托泊苷100 mg/m^2^ d1-d3，静脉滴注；顺铂75 mg/m^2^ d1，静脉滴注；21 d为1个周期。放疗期间依托泊苷维持剂量不变，为100 mg/m^2^ d1-d3，顺铂采用剂量递增（第1组为顺铂70 mg/m^2^ d1，静脉滴注；第2组为顺铂75 mg/m^2^ d1，静脉滴注）。化疗期间给予止吐、水化、保肝等对症处置。化疗前后每周复查血常规和肝肾功能，对出现的骨髓抑制及消化道反应及时对症处置。

放疗采用瓦里安直线加速器，6 MV-10 MV X线。所有患者均采用胸部增强计算机断层扫描（computed tomography, CT）进行定位扫描，扫描层厚为5 mm；扫描范围为下颌骨下缘至肝下缘。CT扫描资料传送至治疗计划系统进行靶区勾画。大体靶体积（gross target volume, GTV）定义为影像学可见的原发病灶及转移淋巴结。原发灶临床靶体积（clinical target volume, CTV）为原发灶GTV的基础上外括8 mm，之后再根据解剖边界进行调整；淋巴结CTV为治疗前的阳性淋巴结引流区。运动靶体积（internal target volume, ITV）为CTV基础上根据呼吸动度进行调整。计划靶体积（planning target volume, PTV）为ITV基础上外括5 mm。放疗处方剂量为45 Gy/30 f，1.5 Gy/f，每日两次，同一日两次放疗间隔时间≥6 h，5天/周，共3周完成。对于放化疗结束后评效达到完全缓解（complete response, CR）或接近CR的患者，在治疗全部结束后4周进行脑预防性放疗（prophylactic cranial irradiation, PCI），剂量为全脑25 Gy/10 f，1次/天，5次/周。

### 1.3 评效及毒副反应评价

治疗结束后1个月进行评效，评效检查包括体格检查、胸部增强CT、腹部超声或CT、颈部淋巴结超声、头MRI、骨扫描。疗效评价根据实体瘤评效标准1.1版本（Response Evaluation Criteria in Solid Tumors, RECIST 1.1）^[5]^分为：完全缓解（complete response, CR）、部分缓解（partial response, PR）、稳定（stable disease, SD）和进展（progressive disease, PD）。以CR+PR计算客观反应率（objective response rate, ORR）。治疗期间相关毒性按照国际癌症组织常见不良反应标准（National Cancer Institute Common Terminology Criteria for Adverse Events, NCI-CTCAE）4.0^[6]^进行评价，每周评价一次。本研究的主要观察指标为放化疗期间的血液学毒性。MTD为6例患者中不超过1例患者出现剂量限制毒性（4级血液学毒性）的剂量，同时下一剂量组6例患者至少2例出现剂量限制毒性。出现4级以上毒性应为剂量限制毒性。次要研究目的为放化疗期间非血液学毒性及生存。

### 1.4 随访

治疗结束后1月进行随访，之后2年内每3个月随访一次，2年-5年每6个月随访一次，5年后每年随访一次。随访内容包括体格检查、血常规、血生化、肿瘤标志物及影像学检查（包括胸部CT、腹部超声/CT、颈部淋巴结超声、头磁共振成像（magnetic resonance imaging, MRI）和骨扫描，其中前5年全身骨扫描每6个月进行一次）。

### 1.5 统计学方法

所有数据资料采用SPSS 19.0软件进行处理。总生存期（overall survival, OS）定义为从诊断日期开始至任何原因所致死亡日期或末次随访日期。无进展生存期（progression-free survival, PFS）定义为从诊断日期开始至疾病进展或末次随访日期。局部无复发生存期（loco-regional failure-free survival, LRFFS）定义为从诊断日期开始至局部失败或末次随访日期。研究采用*Kaplan*-*Meier*法分析OS、PFS和LRFFS。

## 2 结果

### 2.1 剂量递增完成情况

研究共纳入20例患者，其中第1组入组6例患者，第2剂量水平共入组14例患者。在第1组中，有1例患者出现4度血液学毒性，后递增至第2组，其中有1例患者出现4度血液学毒性，1例患者因乏力明显放疗完成27次后停止，1例患者拒绝PCI。其他患者均根据治疗计划完成治疗。

### 2.2 患者临床特点

2013年1月-2016年8月，研究共纳入20例符合条件的LS-SCLC患者，其中第1组入组6例患者，第2组共入组14例患者。患者的临床特点详见表 1。20例患者中，男性15例，女性5例。中位年龄49.50（30-68）岁。70%的患者有吸烟史，10%的患者有肿瘤家族史。Ⅲa期患者占35%，Ⅲb期患者占50%。

**1:** 局限期小细胞肺癌患者临床特征 Clinical characteristics of patients with limited-stage small cell lung cancer

Patient characteristics	Dose level		All patients
	Dose level Ⅰ	Dose level Ⅱ	
Number	6	14	34
Age (yr) [Median age (range)]	49 (43-56)	51.5 (30-68)	49.50 (30-68)
Gender			
Male	5	11	15
Female	1	4	5
History of smoking			
Yes	5	9	14
No	1	5	6
History of cancer			
Yes	1	1	2
No	5	13	18
ECOG performance			
0	4	5	11
1	2	9	9
T stage			
1	0	5	4
2	3	4	7
3	0	1	1
2	5	4	8
N stage			
0	0	1	1
1	0	3	3
2	2	7	10
3	4	3	6
AJCC stage			
Ⅱa	0	3	3
Ⅱb	0	0	0
Ⅲa	2	5	7
Ⅲb	4	6	10
Induction chemotherapy			
Yes	4	11	15
No	2	3	5
ECOG: Eastern Cooperative Oncology Group.			

### 2.3 放化疗相关不良反应

治疗相关毒性具体见表 2。血液学毒性方面，中性粒细胞减少发生率为100%；第1组6例患者中，1例患者出现4度中性粒细胞减低，4例患者出现≥3度血液学毒性；第2组14例患者中，1例患者出现4度中性粒细胞减低和白细胞减低，2例患者出现3度血小板减低，10例患者出现≥3度血液学毒性。发生≥2度以上血液学毒性患者经给予粒细胞集落刺激因子和/或白介素-11对症处理后血象恢复正常。非血液学毒性方面，第1组6例患者中，5例患者出现放射性食管炎发生，其中，1例患者出现3度放射性食管炎，4例患者出现2度放射性食管炎；放射性肺炎发生率为0%。此外，3例患者出现≥2度乏力。第2组14例患者中，7例患者（50.0%）出现放射性食管炎，其中，5例患者（35.7%）出现2度放射性食管炎，无患者出现3度及以上放射性食管炎。2例患者（14.3%）出现放射性肺炎，均为1度放射性肺炎，无患者出现2度及以上放射性肺炎。此外，2例患者（14.3%）出现≥2度乏力。无患者因放化疗相关毒性导致死亡。

**2:** 局限期小细胞肺癌患者治疗相关急性不良反应[*n* (%)] Treatment-related toxicities of patients with limited-stage small cell lung cancer [*n* (%)]

	Grade 0			Grade 1			Grade 2			Grade 3			Grade 4				Grade 5
	Arm Ⅰ	Arm Ⅱ		Arm Ⅰ	Arm Ⅱ		Arm Ⅰ	Arm Ⅱ		Arm Ⅰ	Arm Ⅱ		Arm Ⅰ	Arm Ⅱ		Arm Ⅰ	Arm Ⅱ
Hematological toxicities																	
Leukemia	0 (0.0)	0 (0.0)		0 (0.0)	0 (0.0)		3 (50.0)	5 (35.7)		3 (50.0)	8 (57.1)		0 (0.0)	1 (7.1）			
Neutropenia	0 (0.0)	1 (7.1)		1 (16.7)	0 (0.0)		1 (16.7)	4 (28.6)		3 (50.0)	8 (57.1)		1 (16.7)	1 (7.1)			
Anemia	2 (33.3)	3 (21.4)		2 (33.3)	8 (57.1)		1 (16.7)	3 (21.4)		1 (16.7)	0 (0.0)		0 (0.0)	0 (0.0)			
Thrombocytopenia	3 (50.0)	4 (28.6)		1 (16.7)	4 (28.6)		2 (33.3)	2 (28.6)		0 (0.0)	2 (14.3)		0 (0.0)	0 (0.0)			
Non-hematological toxicities																	
Fatigue	0 (0.0)	1 (7.1)		3 (50.0)	11 (78.6)		2 (33.3)	2 (14.3)		1 (16.7)	0 (0.0)		0 (0.0)	0 (0.0)		0 (0.0)	0 (0.0)
Pneumonia	6 (100.0)	12 (85.7)		0 (0.0)	2 (14.3)		0 (0.0)	0 (0.0)		0 (0.0)	0 (0.0)		0 (0.0)	0 (0.0)		0 (0.0)	0 (0.0)
Esophagitis	1 (16.7)	7 (50.0)		0 (0.0)	2 (14.3)		4 (66.7)	5 (35.7)		1 (16.7)	0 (0.0)		0 (0.0)	0 (0.0)		0 (0.0)	0 (0.0)
Nausea	3 (50.0)	6 (42.9)		3 (50.0)	6 (42.9)		0 (0.0)	2 (14.3)		0 (0.0)	0 (0.0)		0 (0.0)	0 (0.0)		0 (0.0)	0 (0.0)
Vomiting	6 (100.0)	8 (57.1)		0 (0.0)	4 (28.6)		0 (0.0)	2 (14.3)		0 (0.0)	0 (0.0)		0 (0.0)	0 (0.0)		0 (0.0)	0 (0.0)
Hepatic	4 (66.7)	11 (78.6)		2 (33.3)	1 (7.1)		0 (0.0)	1 (7.1)		0 (0.0)	1 (7.1)		0 (0.0)	0 (0.0)		0 (0.0)	0 (0.0)
Hyperbilirubinemia	5 (83.3)	11 (78.6)		0 (0.0)	3 (21.4)		0 (0.0)	0 (0.0)		0 (0.0)	0 (0.0)		0 (0.0)	0 (0.0)		0 (0.0)	0 (0.0)

### 2.4 近期疗效和生存

放化疗完成后20例患者均可评估疗效。有3例患者（15%）达到CR，17例患者（85%）达到PR，总有效率达到100%。中位随访时间为9.0个月（3.2个月-36.2个月），1年生存率、无进展生存期分别为91%、62%。生存结果见图 1、图 2。

**1:**
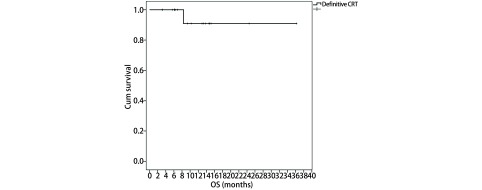
局限期小细胞肺癌患者行每日两次放疗联合EP方案化疗的OS The overall survival (OS) of all patients in limited-stage small cell lung cancer treated with twice-daily radiotherapy and chemotherapy of etoposide and cisplatin

**2:**
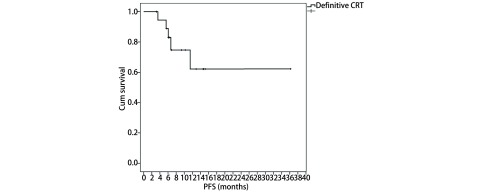
局限期小细胞肺癌患者行每日两次放疗联合EP方案化疗的PFS The progression-free survival (PFS) of all patients in limited-stage small cell lung cancer treated with twice-daily radiotherapy and chemotherapy of etoposide and cisplatin

### 2.5 复发情况

截至末次随访时间，20例患者中有5例患者出现了复发，其中2例患者出现局部复发，3例患者出现远处转移，1例患者出现局部复发和远处转移。最常见的远处转移为脑（10%），其次为肝（5%）、骨（5%）。其中，有1例患者因为拒绝PCI，结束治疗随访10个月后出现了脑转移。

## 3 讨论

目前国内尚无LS-SCLC同步放化疗中EP方案最佳化疗剂量的研究，本研究的初步结果显示依托泊苷100 mg/m^2^ d1-d3联合顺铂75 mg/m^2^ d1，21 d为1个周期联合放疗治疗LS-SCLC是安全可行的。

本研究依据国内常用的化疗方案推荐剂量设定顺铂目标剂量为75 mg/m^2^，由70 mg/m^2^开始剂量递增，观察其安全性和有效性。研究共入组20例患者，中性粒细胞减少是最常见的不良反应，70 mg/m^2^剂量组≥3级中性粒细胞减少发生率为66.7%，75 mg/m^2^剂量组≥3级中性粒细胞减少发生率为71.4%，且4级中性粒细胞减少发生率较低，为7.1%。国外不少研究中采用的是依托泊苷100 mg/m^2^ d1-d3+顺铂80 mg/m^2^ d1，21 d为1个周期的方案。INT 0096研究^[7]^中每日两次放疗组≥3级血液学毒性发生率为87%，其中4级血液学毒性发生率为62%，较此研究血液学毒性明显增加。非血液学毒性方面，INT 0096研究中放射性食管炎发生率为32%，较此研究明显增加，考虑与本研究采用调强放疗技术有关。日本一项Ⅱ期研究^[8]^中，对LS-SCLC患者进行同步放化疗，放疗采用每日两次方案（单次剂量1.5 Gy，总剂量45 Gy），同步化疗采用依托泊苷100 mg/m^2^ d1-d3，顺铂80 mg/m^2^ d1，21 d为1个周期。结果发现有84%患者出现4度中性粒细胞减少，31%出现中性粒细胞减少伴发热，其血液学毒性较此研究也明显增加。国内王慧娟等^[9]^开展的一项广泛期小细胞肺癌的多中心临床研究进行伊立替康联合顺铂与依托泊苷联合顺铂的比较中推荐的EP方案剂量具体为依托泊苷100 mg/m^2^ d1-d3，顺铂25 mg/m^2^ d1-d3，21 d为1个周期，结果Ⅲ度以上粒细胞减少发生率为61.5%，Ⅲ度以上血小板降低为3.8%，与本研究结果相似。

本研究完成率较高，20例患者仅有1例患者因治疗相关毒性未能完成放疗，而日本的Ⅱ期研究^[8]^入组51例患者，采用顺铂80 mg/m^2^的剂量，有6例患者因治疗相关毒性引起治疗延迟，与之相比，本研究中顺铂75 mg/m^2^的剂量是较安全的。此外，本研究的中位生存期和中位无进展生存期尚未达到，中位随访9.0个月，1年OS为91%，1年PFS为62%。虽然由于研究入选病例的时段较长造成观察时间差异较大，中位随访时间较短，但从1年生存率来看，此结果与其他研究^[7, 10]^相比，也是具有可比性的。因而LS-SCLC同步放化疗中，将顺铂化疗剂量递增至75 mg/m^2^，治疗相关毒性可耐受，患者放化疗不易中断，进而有可能进一步提高局部控制率和生存。

脑转移是小细胞肺癌最常见的失败模式。多项研究^[11-13]^显示，小细胞肺癌治疗后2年脑转移发生率约为50%。此研究截至末次随访时间，脑转移发生率为10%，较低，可能与随访时间过短有关，需要更长的随访时间来证实。

综上所述，LS-SCLC患者采用加速超分割放疗联合EP方案化疗将顺铂剂量递增至75 mg/m^2^是安全的，其有效性还需要进一步扩大样本量和随访更长的时间来证实。
